# Bidirectional Mendelian Randomization Analysis of the Association Between Mitochondrial Proteins and Neurodegenerative Diseases

**DOI:** 10.1002/brb3.70283

**Published:** 2025-01-20

**Authors:** Fangyuan Wang, Zhou Jing, Qingyi Wang, Minghe Li, Bingqi Lu, Ao Huo, Chenglin Zhao, Huanyu Zhou, Wulong Liang, Weihua Hu, Xudong Fu

**Affiliations:** ^1^ Department of Neurosurgery, The Fifth Affiliated Hospital of Zhengzhou University Zhengzhou University Zhengzhou China; ^2^ Department of Clinical Medicine, The First Clinical Medical College of Zhengzhou University Zhengzhou University Zhengzhou China

**Keywords:** mitochondrial proteins, neurodegenerative diseases, mendelian randomization analysis

## Abstract

**Background:**

Neurodegenerative diseases involve progressive neuronal dysfunction and cognitive decline, posing substantial global challenges. Although the precise causes remain unclear, several studies highlight the role of protein metabolism abnormalities in disease development. This study investigates the causal links between variations in mitochondrial protein genes and neurodegenerative diseases, aiming to elucidate their potential contributions to disease progression and identify novel therapeutic strategies.

**Methods:**

Herein, we utilized data from genome‐wide association studies (GWAS) on mitochondrial proteins and neurodegenerative diseases. Bidirectional Mendelian randomization (MR), employing instrumental variables (IVs), was used to assess causal relationships. The primary method for estimating causal effects was the inverse variance‐weighted (IVW) method, supplemented by additional MR approaches.

**Results:**

Bidirectional MR revealed significant associations between mitochondrial protein gene variants and neurodegenerative diseases. Specifically, associations were found with Alzheimer's disease (AD) (three proteins), Parkinson's disease (PD) (four proteins), amyotrophic lateral sclerosis (ALS) (six proteins), multiple sclerosis (two proteins), and dementia with Lewy bodies (four proteins). Conversely, analyses indicated significant associations of neurodegenerative diseases with mitochondrial protein gene variants, notably with AD, dementia with Lewy bodies, and multiple sclerosis, affecting multiple mitochondrial protein levels. Bidirectional causality was observed between dementia with Lewy bodies and C21orf33.

**Conclusions:**

Using MR, we identified significant links between mitochondrial protein gene mutations and the risk of neurodegenerative diseases. These results highlight reciprocal relationships where certain neurodegenerative diseases influence mitochondrial protein expression levels. These findings underscore the pivotal role of mitochondrial proteins in neurodegenerative diseases, offering critical insights into disease mechanisms and potential therapeutic avenues.

## Graphical abstract

We utilized data from genome‐wide association studies (GWAS) on mitochondrial proteins and neurodegenerative diseases. Bidirectional Mendelian randomization (MR), employing instrumental variables (IVs), was used to assess causal relationships. The primary method for estimating causal effects was the inverse variance‐weighted (IVW) method, supplemented by weighted median, simple median, MR‐PRESSO, and MR‐Egger. Using MR, we identified significant links between mitochondrial protein gene mutations and the risk of neurodegenerative diseases. These results highlight reciprocal relationships where certain neurodegenerative diseases influence mitochondrial protein expression levels. These findings underscore the pivotal role of mitochondrial proteins in neurodegenerative diseases, offering critical insights into disease mechanisms and potential therapeutic avenues.

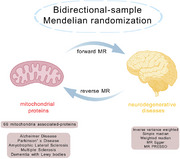

## 1 Introduction

Neurodegenerative diseases, such as Alzheimer's disease (AD), Parkinson's disease (PD), amyotrophic lateral sclerosis (ALS), and Huntington's disease, present significant global health challenges owing to their progressive and debilitating nature (Alzheimer's Association 2020; Poewe et al. 2017; Brown and Al‐Chalabi 2017; Ross and Tabrizi 2011). These conditions are marked by the gradual loss of neuronal structure and function, resulting in severe cognitive and motor impairments that drastically reduce quality of life and increase healthcare costs. As the global population ages, the incidence and prevalence of these diseases are expected to increase, thus emphasizing the need for a deeper understanding of the underlying mechanisms to develop effective interventions.

Mitochondria, distinguished by their unique nonnuclear DNA (Abuarab et al. 2017), are essential for cellular physiology, particularly in neurons, as they are the primary energy source. These organelles are pivotal for maintaining normal brain function and metabolism. Emerging evidence has linked mitochondrial dysfunction to the pathogenesis of neurodegenerative diseases; this dysfunction manifests as mitochondrial damage, genetic mutations, impaired dynamics, altered calcium homeostasis, and diminished bioenergetics. Such disruptions in mitochondrial function are now recognized as key contributors to the progression of neurodegenerative disorders (Area‐Gomez and Schon 2016; Pickrell and Youle 2015; Swerdlow and Khan 2004).

Genetic studies have previously highlighted the association between mutations in genes encoding mitochondrial proteins and the risk of developing neurodegenerative diseases. Mutations in genes such as PINK1 and Parkin are associated with familial PD, underscoring the significance of mitochondrial quality control mechanisms in disease development. These genetic insights indicate that mitochondrial proteins play a crucial role in maintaining neuronal health and preventing neurodegeneration (Bonello et al. 2019, Calabresi and Ghiglieri 2014, Tripathi et al. 2024, Yi et al. 2019). However, traditional correlational studies face challenges in establishing causality, specifically whether genetic variations cause the disease or whether the disease state influences gene expression.

Mendelian randomization (MR) provides a robust methodological approach to address this issue, using genetic variants as instrumental variables (IVs) to infer causal relationships (Lawlor et al. 2008). Bidirectional MR analysis, which uses genetic variants as IVs to estimate causal relationships between exposure and outcomes, provides a fresh perspective on this issue.

The present study aimed to explore the causal relationships and potential therapeutic implications of mitochondrial protein gene variations in neurodegenerative diseases using bidirectional MR analysis.

## 2 Materials and Methods

### 2.1 Data Sources

Mitochondrial protein data were obtained from the genome‐wide association study (GWAS) catalog (https://www.ebi.ac.uk/gwas/), sourced from the Genomic Atlas of the Human Plasma Proteome (PMID: 29875488) (Sun et al. 2018). The dataset comprises 3301 European samples and 3283 proteins. Among these, 66 proteins related to mitochondria were identified (Table S1). Neurodegenerative disease‐related GWAS data (Table S2) were sourced from IEU OpenGWAS (https://gwas.mrcieu.ac.uk/). These databases collected ethical approval and informed consent, as such, no additional explanations were required.

### 2.2 Selection of IVs

When conducting MR analysis through IV selection, three key assumptions must be met. First, the relevance assumption stipulates that the IV (SNP) must exhibit a significant association with exposure, with the *F*‐value considered a measure of this association. Second, the independence assumption requires that the IV be independent of confounding factors, meaning that it should not directly influence the outcome, unless through the exposure factor. Third, the exclusion restriction assumption states that the impact of IV on the outcome must be entirely mediated through the exposure factor, not through any other independent pathways (Didelez and Sheehan 2007, Zhu et al. 2023).

On the basis of the previous stipulations, we constructed genetic IVs for mitochondrial proteins and neurodegenerative disease‐related traits based on the following criteria: In the MR analysis, we employed the following standards to select IVs: (a) linkage disequilibrium (LD), measured by an *r* ^2^ less than 0.001 between IVs within a 10,000 kb window. LD refers to the tendency of genetic variants close in genomic position to be inherited together, potentially biasing effect estimates when variants are correlated; (b) IVs with *p* values below genome‐wide significance level (5 × 10^−6^), indicating their association with exposure in GWAS studies; (c) minor allele frequency (MAF) of IVs greater than 0.01; (d) IVs with an *F*‐statistic greater than 10, indicating a strong correlation (Salehi Nowbandegani et al. 2023).

### 2.3 MR Analysis

MR analysis typically uses the inverse variance‐weighted (IVW) method, which is one of the most common and widely used approaches. The IVW method employs a meta‐analysis approach that combines ratio estimates from individual SNPs, weighted by their inverse variances, to estimate the impact of risk factors on outcomes. Ratio estimates involve dividing the effect of a single SNP on the outcomes by its effect on risk factors, assuming that all associations are log‐linear. When all IVs are valid, the IVW method provides reliable estimates that meet the core assumptions of MR analysis (Pairo‐Castineira et al. 2023).

There are several other methods for assessing the correlation between genes and outcomes, such as the weighted median, simple median, MR‐PRESSO [15], and MR‐Egger. In these methods, a significance level of *p* < 0.05 is typically considered statistically significant. The weighted median and simple median methods demonstrated a higher tolerance to pleiotropic genetic variations, providing relatively stable effect estimates, even when up to half of the IVs were invalid. The primary distinction between these methods lies in their handling of median estimation. The simple median method assigns equal weights to all values, whereas the weighted median method assigns different weights to each value (Bowden et al. 2017). The MR‐PRESSO method is useful in detecting and correcting horizontal pleiotropy, identifying outlier genetic variants, and assessing differences in outcomes before and after correction (Verbanck et al. 2018).

### 2.4 Tests for Horizontal Pleiotropy and Heterogeneity

Tests for horizontal pleiotropy and heterogeneity typically utilize MR‐Egger regression and Cochran's *Q* test. In this study, MR‐Egger regression was applied to estimate horizontal pleiotropy, whereas Cochran's *Q* test was used to assess heterogeneity. We excluded the possibility of an MR‐Egger intercept *p* value less than 0.05 and addressed potential horizontal pleiotropy. If Cochran's *Q* test yielded a *p* value of less than 0.05, the final MR results will reference the IVW multiplicative random effects model. In addition, we conducted a leave‐one‐out sensitivity analysis to evaluate the independent strength of each IV. We considered *p* values less than 0.05 indicative of statistically significant genetic associations between exposure and outcome.

### 2.5 Reverse MR

Genetic IVs for mitochondrial proteins and neurodegenerative disease‐related traits were constructed on the basis of the following criteria in MR analysis: (a) LD measured by an *r* ^2^ less than 0.01 between IVs within a 5000 kb window. LD refers to the tendency of genetic variants close in genomic position to be inherited together, potentially biasing effect estimates; (b) IVs with *p* values below genome‐wide significance level (5 × 10^−6^), indicating their association with exposure in GWAS studies; (c) MAF of IVs greater than 0.01; (d) IVs with an *F*‐statistic greater than 10, indicating strong correlation.

### 2.6 Statistical Analysis

All two‐sided statistical analyses were determined using a threshold of *p* < 0.05 for statistical significance. These analyses were performed using R version 4.2.0, using the packages “ieugwasr,” “TwoSampleMR,” “MRPRESSO,” and “gtx.”

## 3 Results

### 3.1 Mitochondrial Protein to AD MR Analysis

**FIGURE 1:**
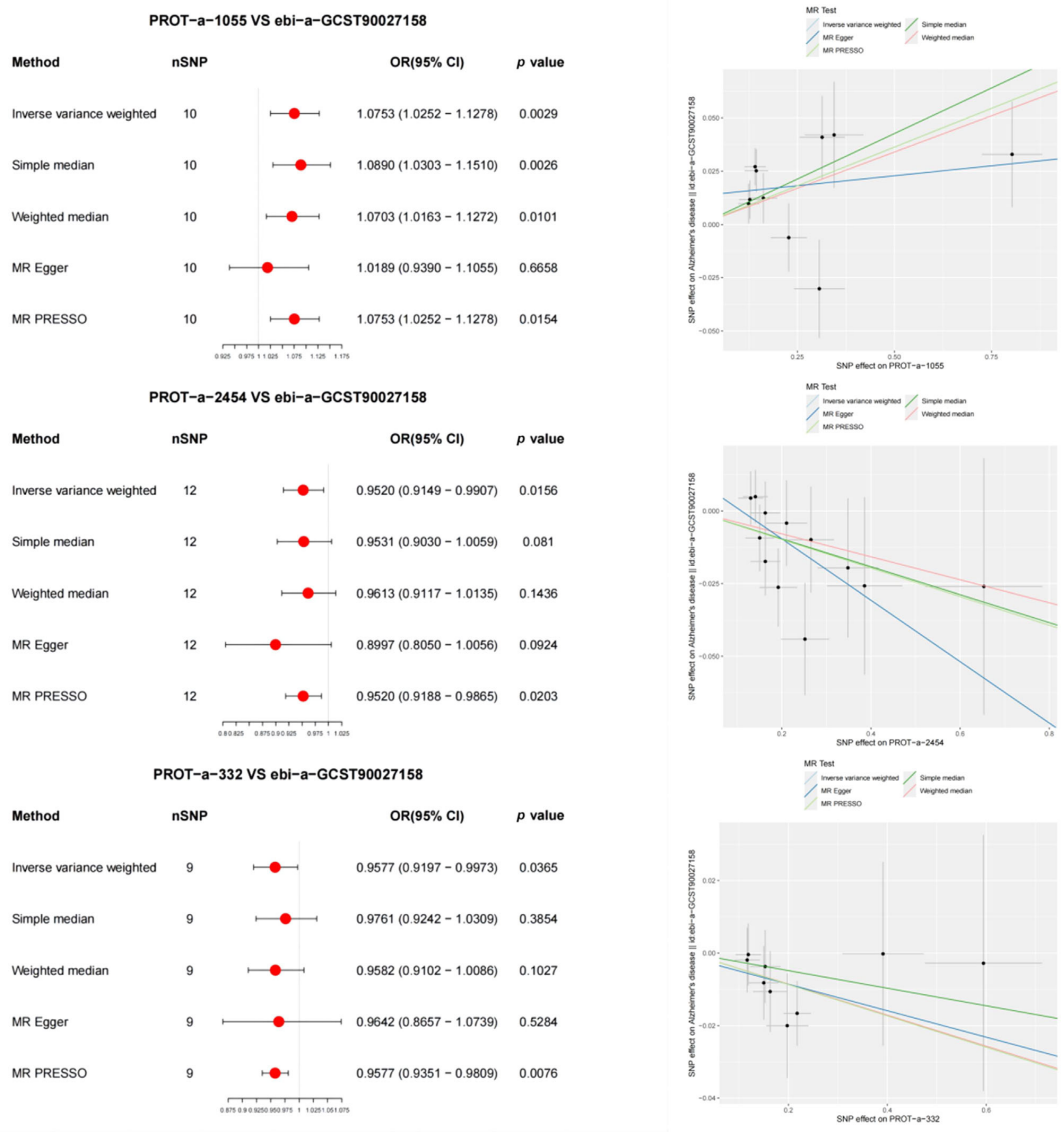
Forest and scatter plots of the Mendelian randomization (MR) analysis results, indicating a significant negative correlation between mitochondrial proteins and AD, as well as neurodegenerative diseases. OD, odds ratio.

In the forward MR analysis, the results indicated significant associations between AD and 3 out of 66 mitochondrial proteins (Figure 1). In particular, PROT‐a‐1055 (FARS2) was notably associated with AD (IVW: odds ratio [OR] = 1.0753, 95% CI 1.0252–1.1278, *p* = 0.0029). MR‐PRESSO detected consistent correlations between mitochondrial proteins and AD compared to the IVW results. The MR‐PRESSO global test results revealed no evidence of directional pleiotropy for PROT‐a‐1055 (FARS2) or AD (*p* = 0.105).

Additionally, lower levels of PROT‐a‐2454 (TRUB1) and PROT‐a‐332 (CA5A) were associated with AD (IVW: OR = 0.9520, 95% CI 0.9149–0.99070, *p* = 0.0156; IVW: OR = 0.9577, 95% CI 0.9197–0.9973, *p* = 0.0365). The MR‐PRESSO analysis corroborated these findings, with results consistent with those of IVW. MR‐PRESSO global tests showed no directional pleiotropy for these two proteins or for AD (*p* = 0.654 and 0.934, respectively).

**FIGURE 2:**
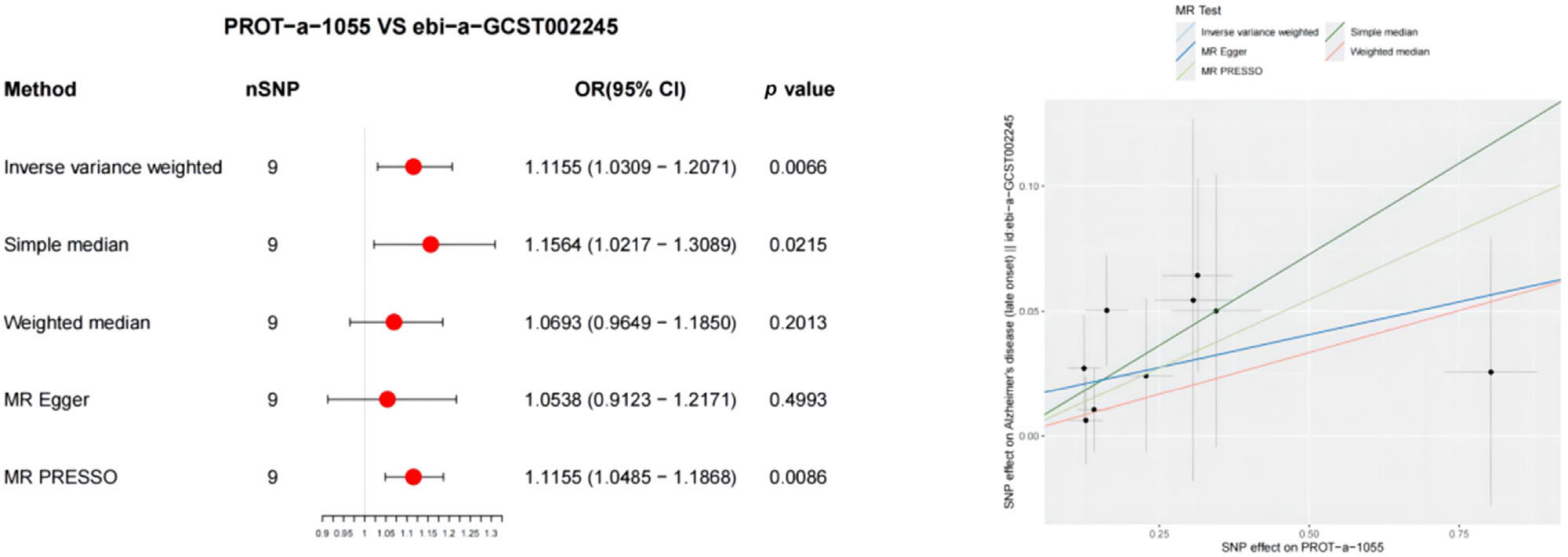
Forest and scatter plots of the Mendelian randomization (MR) analysis results, illustrating a significant negative correlation between PROT‐a‐1055 (FARS2) and AD (validated), as well as a significant correlation with neurodegenerative diseases. OD, odds ratio.

The significant association between PROT‐a‐1055 (FARS2) and AD was further validated using the ieu‐b‐2 dataset (IVW: OR = 1.0753, 95% CI 1.0252–1.1278, *p* = 0.0029) (Figure 2). MR‐PRESSO confirmed consistent correlations between mitochondrial proteins and AD compared to the IVW results. The MR‐PRESSO global test results showed no directional pleiotropy for PROT‐a‐1055 (FARS2) or AD (*p* = 0.699).

### 3.2 Mitochondrial Protein to PD MR Analysis

**FIGURE 3:**
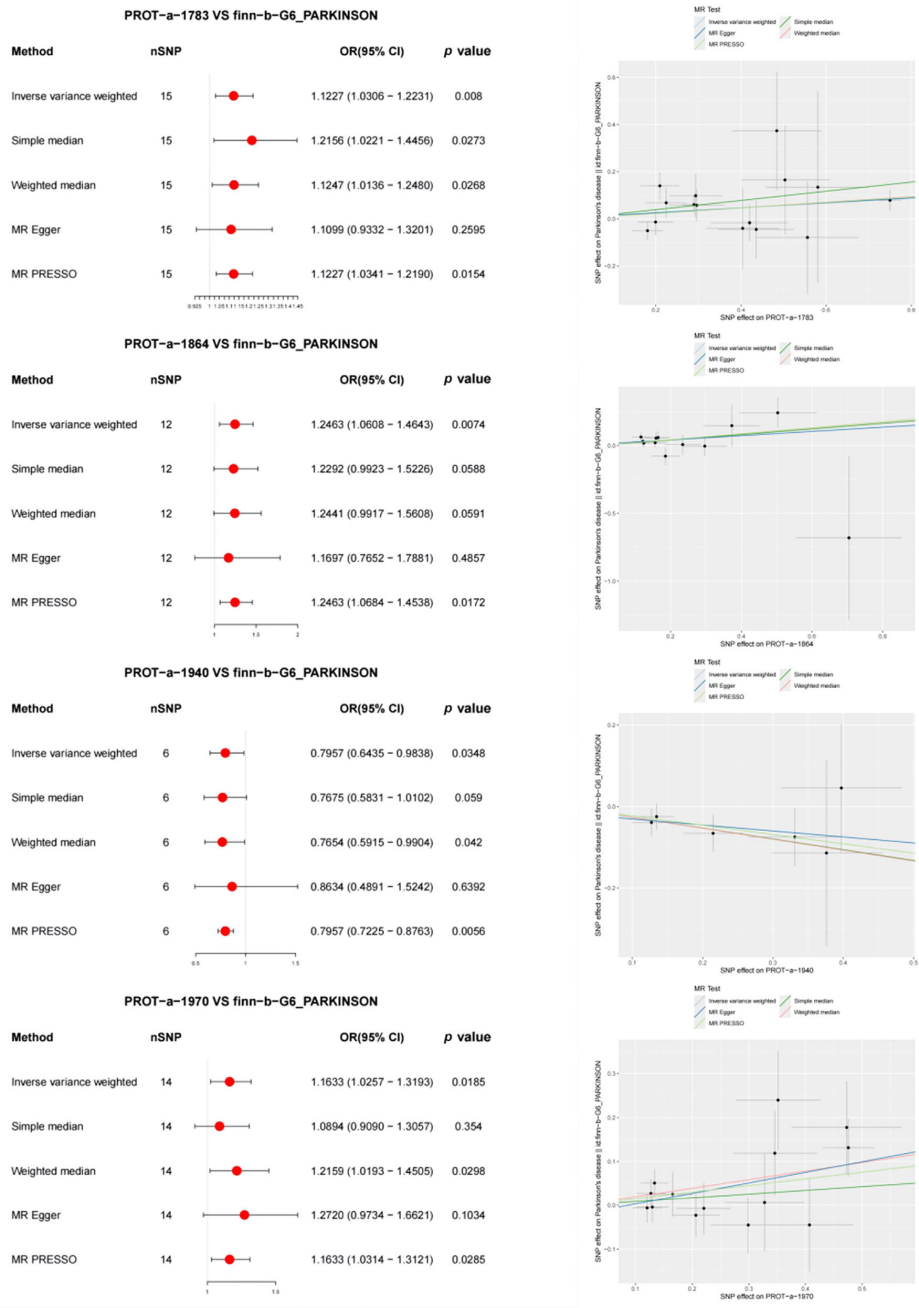
Forest and scatter plot of the Mendelian randomization (MR) analysis results, showing a significant negative correlation between mitochondrial proteins and PD. OD, odds ratio.

In the forward MR analysis, the results indicated significant associations between PD and 4 out of 66 mitochondrial proteins (Figure 3). Specifically, PROT‐a‐1783 (LRPPRC), PROT‐a‐1864 (MCEE), and PROT‐a‐1970 (MUL1) showed notable associations with PD (IVW: OR = 1.1227, 95% CI 1.0306–1.2231, *p* = 0.008; IVW: OR = 1.2463, 95% CI 1.0608–1.4643, *p* = 0.0074; IVW: OR = 1.1633, 95% CI 1.0257–1.3193, *p* = 0.0185). MR‐PRESSO further detected correlations between mitochondrial proteins and PD, which were consistent with the IVW results. The MR‐PRESSO global test results revealed no evidence of directional pleiotropy for PROT‐a‐1055 (FARS2) or PD (*p* = 0.647, *p* = 0.575, and *p* = 0.57).

Additionally, PROT‐a‐1940 (MRPL14) exhibited a weaker association with PD (IVW: OR = 0.7957, 95% CI 0.6435–0.983, *p* = 0.0348). MR‐PRESSO analysis further confirmed the consistency with IVW regarding the correlation between mitochondrial proteins and PD. MR‐PRESSO global tests showed no directional pleiotropy for these two proteins or for PD (*p* = 0.97).

**FIGURE 4:**
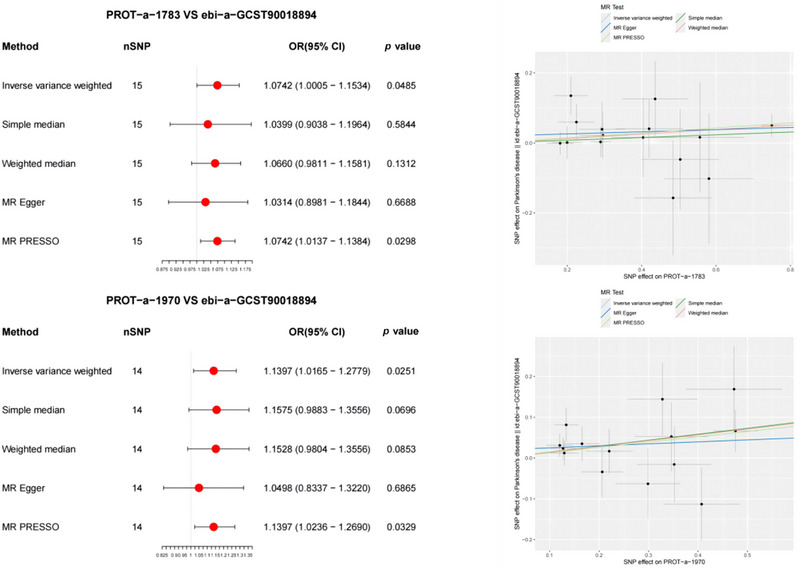
Forest and scatter plot of the Mendelian randomization (MR) analysis results, illustrating a significant negative correlation between mitochondrial proteins and PD (validated), as well as a significant correlation with PD. OD, odds ratio.

The significant associations of PROT‐a‐1783 (LRPPRC) and PROT‐a‐1970 (MUL1) with PD were also validated using the ebi‐a‐GCST90018894 dataset (IVW: OR = 1.0742, 95% CI 1.0005–1.1534, *p* = 0.0485; IVW: OR = 1.1397, 95% CI 1.0165–1.279, *p* = 0.0251) (Figure 4). MR‐PRESSO detected correlations between mitochondrial proteins and PD, which were consistent with the IVW results. The MR‐PRESSO global test results showed no evidence of directional pleiotropy for PROT‐a‐1055 (FARS2) or PD (*p* = 0.872 and 0.616, respectively).

### 3.3 Mitochondrial Protein to ALS MR Analysis

**FIGURE 5:**
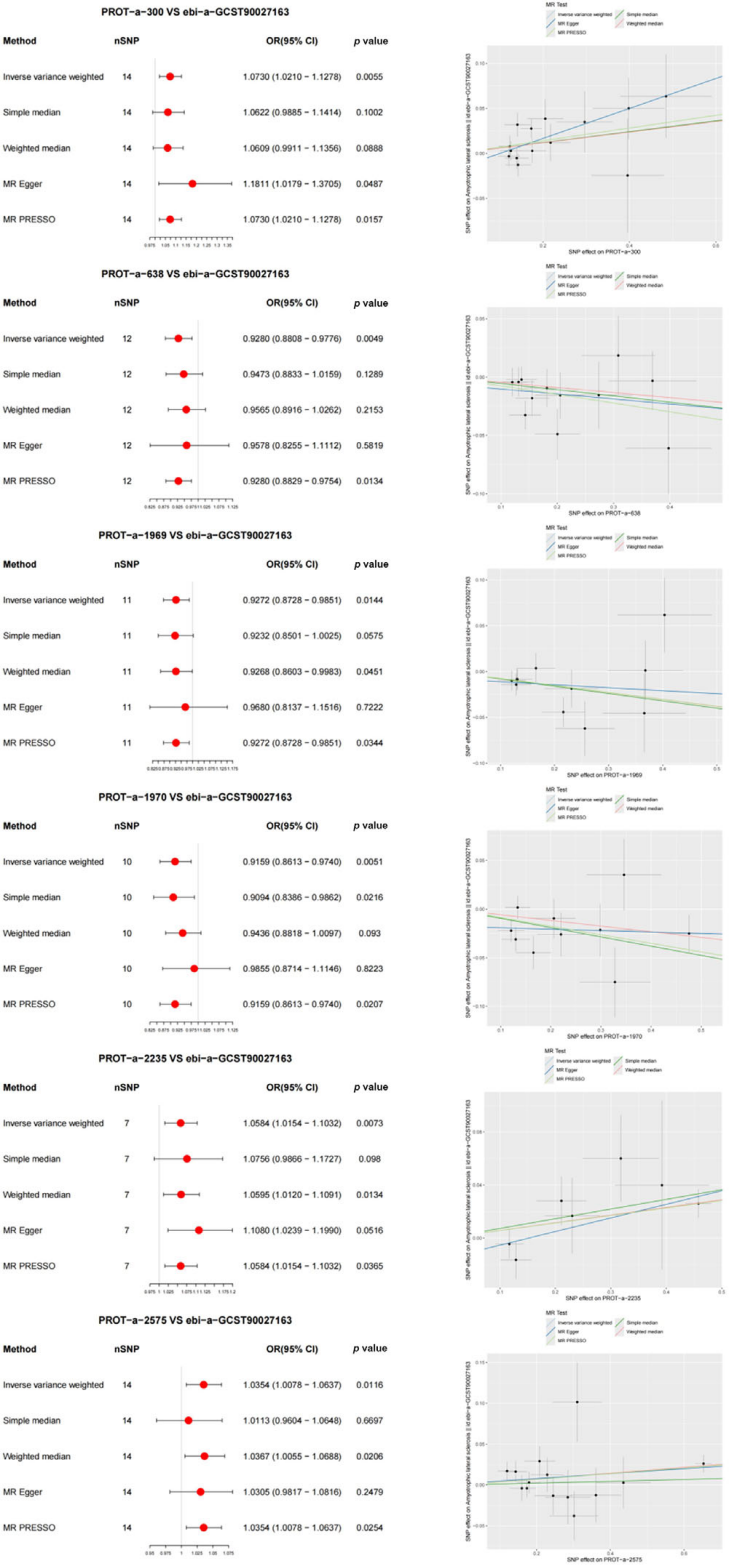
Forest and scatter plot of the Mendelian randomization (MR) analysis results, demonstrating a significant negative correlation between mitochondrial proteins and ALS. OD, odds ratio.

In the forward MR analysis, the results indicated significant associations between ALS and 6 out of 66 mitochondrial proteins (Figure 5). Specifically, PROT‐a‐300 (C1QBP), PROT‐a‐2235 (PDK1), and PROT‐a‐2575 (MRMT3) demonstrated notable associations with ALS (IVW: OR = 1.0730, 95% CI 1.0210–1.1278, *p* = 0.0055; IVW: OR = 1.0584, 95% CI 1.0154–1.1032, *p* = 0.0073; IVW: OR = 1.0354, 95% CI 1.0078–1.0637, *p* = 0.0116). MR‐PRESSO further detected correlations between mitochondrial proteins and ALS, which were consistent with the IVW results. The MR‐PRESSO global test results showed no evidence of directional pleiotropy for PROT‐a‐1055 (FARS2) or ALS (*p* = 0.393, *p* = 0.6 and 0.521, respectively).

Additionally, PROT‐a‐638 (COX5B), PROT‐a‐1969 (MUL1), and PROT‐a‐1970 (MUL1) exhibited lower levels associated with ALS (IVW: OR = 0.9280, 95% CI 0.8808–0.9776, *p* = 0.0049; IVW: OR = 0.9272, 95% CI 0.8728–0.9851, *p* = 0.0144; IVW: OR = 0.9159, 95% CI 0.8613–0.9740, *p* = 0.0051). MR‐PRESSO analysis further confirmed the consistency with IVW regarding the correlation between mitochondrial proteins and ALS. MR‐PRESSO global tests showed no directional pleiotropy for these two proteins and ALS (*p* = 0.566, 0.305, and 0.242, respectively).

**FIGURE 6:**
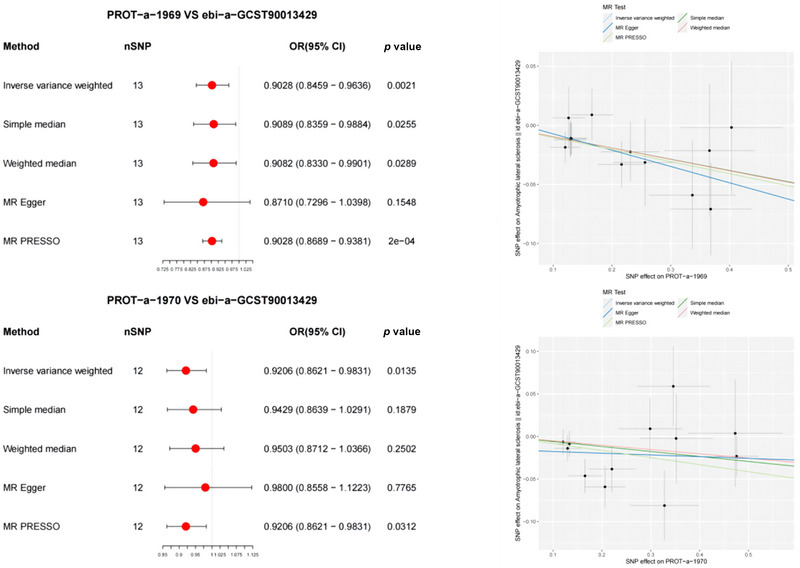
Forest and scatter plot of the Mendelian randomization (MR) analysis results, demonstrating a significant negative correlation between mitochondrial proteins and ALS (validation), as well as a significant correlation between mitochondrial proteins and ALS. OD, odds ratio.

PROT‐a‐1969 (MUL1) and PROT‐a‐1970 (MUL1) were significantly associated with ALS, which was validated using the ebi‐a‐GCST90013429 dataset (IVW: OR = 0.9028, 95% CI 0.8459–0.9636, *p* = 0.0021; IVW: OR = 0.9206, 95% CI 0.8621–0.9831, *p* = 0.0135) (Figure 6). MR‐PRESSO analysis confirmed the consistency of mitochondrial protein correlations with ALS compared to the IVW results. The MR‐PRESSO global tests showed no evidence of directional pleiotropy for PROT‐a‐1055 (FARS2) or ALS (*p* = 0.988 and 0.333, respectively).

### 3.4 Mitochondrial Protein to Multiple Sclerosis MR Analysis

**FIGURE 7:**
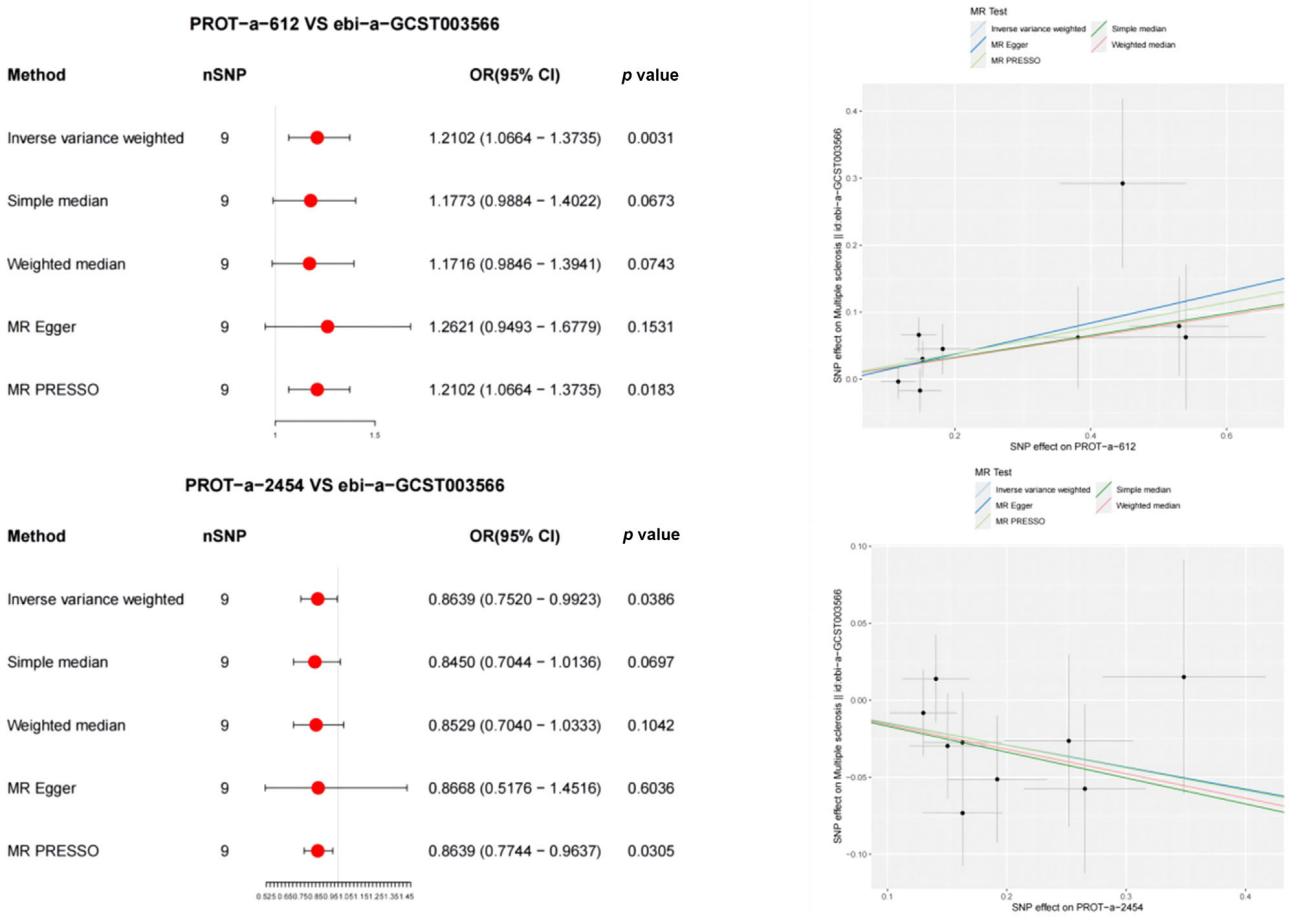
Mendelian randomization (MR) analysis results shown as the forest and scatter plots, showing significant negative correlations between mitochondrial proteins and multiple sclerosis. OD, odds ratio.

In the forward MR analysis, the results showed significant correlations between 2 of the 66 mitochondrial proteins and multiple sclerosis (Figure 7). Among them, a higher level of PROT‐a‐612 (COA3) was associated with the onset of MS (IVW: OR = 1.2102, 95% CI 1.0664–1.3735, *p* = 0.0031). The MR‐PRESSO method confirmed the correlation between mitochondrial proteins and MS, which is consistent with the IVW results. The MR‐PRESSO global test results showed no directional heterogeneity in the association between PROT‐a‐1055 (FARS2) and multiple sclerosis (*p* = 0.488).

Additionally, a lower level of PROT‐a‐2454 (TRUB1) protein was associated with the occurrence of multiple sclerosis (IVW: OR = 0.8639, 95% CI 0.7520–0.9923, *p* = 0.0386). MR‐PRESSO analysis confirmed the consistency between mitochondrial proteins and multiple sclerosis associations with the IVW results. The MR‐PRESSO global test results revealed no directional heterogeneity in the association between PROT‐a‐2454 (TRUB1) and multiple sclerosis (*p* = 0.764).

### 3.5 Mitochondrial Protein to Dementia With Lewy Bodies MR Analysis

**FIGURE 8:**
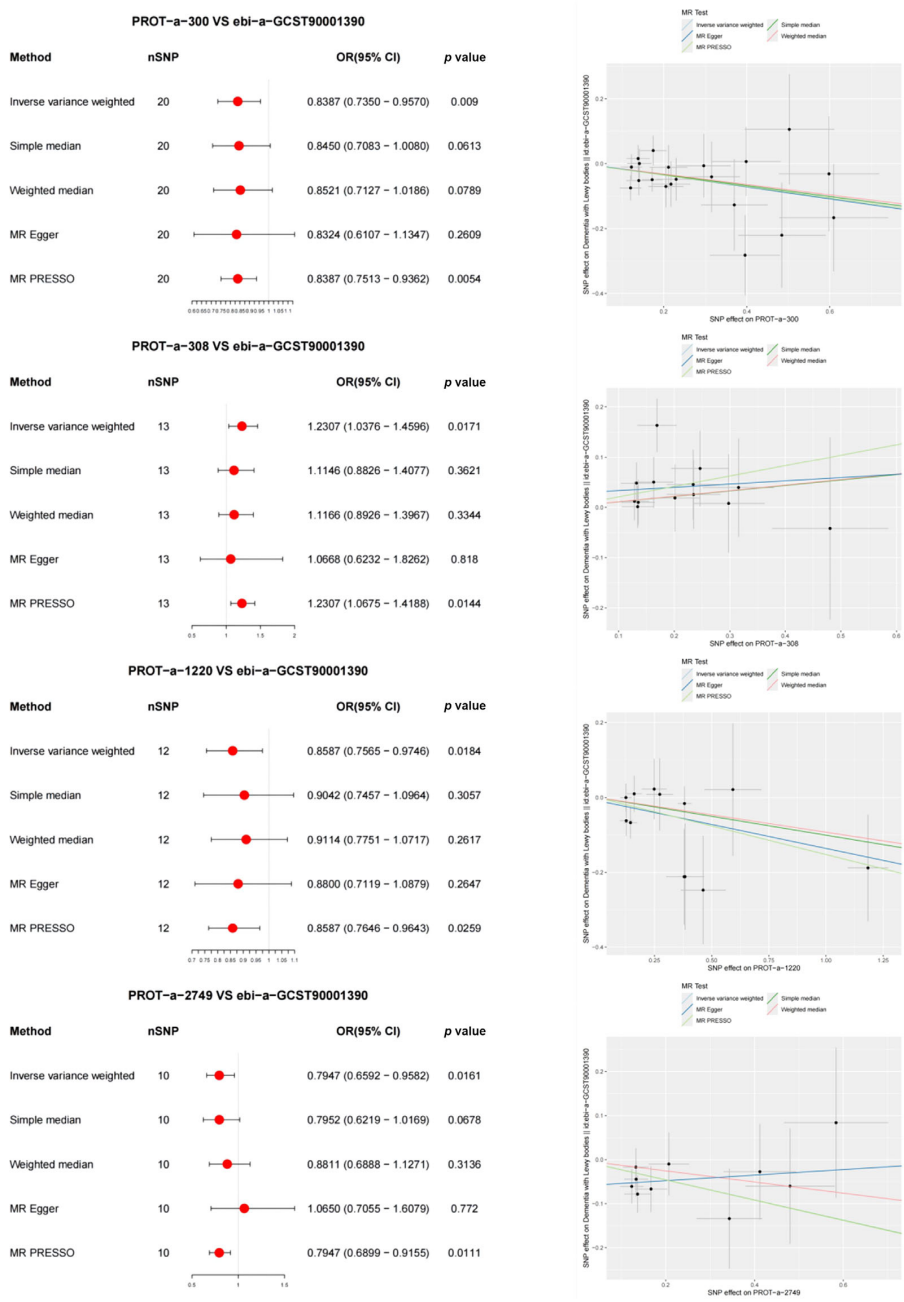
Mendelian randomization (MR) analysis results shown as the forest and scatter plot, showing significant negative correlations between mitochondrial proteins and dementia with Lewy bodies. OD, odds ratio.

Forward MR analysis revealed significant correlations between 4 of 66 mitochondrial proteins and dementia with Lewy bodies (Figure 8). Among them, a higher level of PROT‐a‐308 (C21orf33) was associated with dementia with Lewy bodies onset (IVW: OR = 0.8587, 95% CI 0.7565–0.9746, *p* = 0.0184). The MR‐PRESSO method detected mitochondrial protein correlations with dementia with Lewy bodies, consistent with the IVW results. The MR‐PRESSO global test results showed no directional heterogeneity in the association between PROT‐a‐308 (C21orf33) and dementia with Lewy bodies (*p* = 0.772).

Additionally, lower levels of PROT‐a‐300 (C1QBP), PROT‐a‐1220 (GLRX2), and PROT‐a‐2749 (SLC25A18) proteins were found to be associated with the occurrence of dementia with Lewy bodies (IVW: OR = 0.8387, 95% CI 0.7350–0.9570, *p* = 0.009; IVW: OR = 0.8587, 95% CI 0.7565–0.9746, *p* = 0.0184; IVW: OR = 0.7947, 95% CI 0.6592–0.9582, *p* = 0.0161). MR‐PRESSO analysis confirmed the consistency between mitochondrial proteins and dementia with Lewy bodies associated with IVW results. The MR‐PRESSO global test results showed no directional heterogeneity in the association between these three proteins and dementia with Lewy bodies (*p* = 0.851, 0.611, and 0.836, respectively).

### 3.6 Level Multiplicity and Heterogeneity Tests

The results of the level multiplicity and heterogeneity tests for exposure factors (mitochondrial proteins) and outcome factors (neurodegenerative diseases) were as follows: Egger ’s intercept estimation values (all very close to 0), standard errors, and *p* values (> 0.05). These results indicate that there was no statistically significant multiplicity in this analysis, meaning that the impact of a gene on multiple traits was not significant. The IVW method, Cochran's *Q* statistic, degrees of freedom, and *p* values (*Q*_*p* > 0.05) also indicated that the multiplicity in the studies did not reach statistical significance (Table 1). These results suggest that the relationship between mitochondrial proteins and neurodegenerative diseases was not significantly influenced by other factors.

**TABLE 1:** Level multiplicity and heterogeneity tests between mitochondrial proteins and neurodegenerative diseases.

id.outcome	Exposure	*Q*	*Q*_*p*val	egger_intercept	*p*val
Alzheimer disease	PROT‐a‐1055 (FARS2)	12.1058	0.1465	0.0135	0.1617
Alzheimer disease	PROT‐a‐1055 (FARS2)	15.7034	0.0733		
Alzheimer disease	PROT‐a‐2454 (TRUB1)	7.6372	0.6642	0.0115	0.3115
Alzheimer disease	PROT‐a‐2454 (TRUB1)	8.7733	0.6428		
Alzheimer disease	PROT‐a‐332 (CA5A)	2.7779	0.9048	−0.0013	0.8985
Alzheimer disease	PROT‐a‐332 (CA5A)	2.7954	0.9465		
Alzheimer disease (test)	PROT‐a‐1055 (FARS2)	4.0781	0.7707	0.0144	0.3857
Alzheimer disease (test)	PROT‐a‐1055 (FARS2)	4.9339	0.7646		
Alzheimer disease (test)	PROT‐a‐1965	5.1971	0.5188	−0.0228	0.4531
Alzheimer disease (test)	PROT‐a‐1965	5.8406	0.5585		
Parkinson's disease	PROT‐a‐1783 (LRPPRC)	12.9152	0.4544	0.0051	0.8838
Parkinson's disease	PROT‐a‐1783 (LRPPRC)	12.9375	0.5315		
Parkinson's disease	PROT‐a‐1864 (MCEE)	9.9406	0.4457	0.0115	0.7580
Parkinson's disease	PROT‐a‐1864 (MCEE)	10.0409	0.5267		
Parkinson's disease	PROT‐a‐1940 (MRPL14)	0.9415	0.9185	−0.0159	0.7765
Parkinson's disease	PROT‐a‐1940 (MRPL14)	1.0337	0.9598		
Parkinson's disease	PROT‐a‐1970 (MUL1)	11.3476	0.4994	−0.0209	0.4728
Parkinson's disease	PROT‐a‐1970 (MUL1)	11.8972	0.5361		
Parkinson's disease (test)	PROT‐a‐1783 (LRPPRC)	8.8652	0.7830	0.0198	0.5131
Parkinson's disease (test)	PROT‐a‐1783 (LRPPRC)	9.3174	0.8102		
Parkinson's disease (test)	PROT‐a‐1970 (MUL1)	10.8055	0.5457	0.0203	0.4367
Parkinson's disease (test)	PROT‐a‐1970 (MUL1)	11.4529	0.5729		
Amyotrophic lateral sclerosis	PROT‐a‐1969 (MUL1)	11.9854	0.2141	−0.0079	0.6149
Amyotrophic lateral sclerosis	PROT‐a‐1969 (MUL1)	12.3470	0.2625		
Amyotrophic lateral sclerosis	PROT‐a‐1970 (MUL1)	11.2468	0.1881	−0.0177	0.2203
Amyotrophic lateral sclerosis	PROT‐a‐1970 (MUL1)	13.7322	0.1322		
Amyotrophic lateral sclerosis	PROT‐a‐2235 (PDK1)	4.6068	0.4657	−0.0156	0.2434
Amyotrophic lateral sclerosis	PROT‐a‐2235 (PDK1)	6.3546	0.3847		
Amyotrophic lateral sclerosis	PROT‐a‐2575 (MRMT3)	13.2036	0.3544	0.0019	0.8173
Amyotrophic lateral sclerosis	PROT‐a‐2575 (MRMT3)	13.2649	0.4276		
Amyotrophic lateral sclerosis	PROT‐a‐300 (C1QBP)	12.6125	0.3978	−0.0166	0.2060
Amyotrophic lateral sclerosis	PROT‐a‐300 (C1QBP)	14.4917	0.3401		
Amyotrophic lateral sclerosis	PROT‐a‐638 (COX5B)	9.8356	0.4550	−0.0060	0.6658
Amyotrophic lateral sclerosis	PROT‐a‐638 (COX5B)	10.0336	0.5274		
Multiple sclerosis	PROT‐a‐2454 (TRUB1)	4.9777	0.6627	−0.0006	0.9898
Multiple sclerosis	PROT‐a‐2454 (TRUB1)	4.9779	0.7599		
Multiple sclerosis	PROT‐a‐612 (COA3)	8.0220	0.3307	−0.0092	0.7528
Multiple sclerosis	PROT‐a‐612 (COA3)	8.1450	0.4194		
Dementia with Lewy bodies	PROT‐a‐1220 (GLRX2)	9.1546	0.5175	−0.0082	0.7829
Dementia with Lewy bodies	PROT‐a‐1220 (GLRX2)	9.2347	0.6002		
Dementia with Lewy bodies	PROT‐a‐2749 (SLC25A18)	2.7076	0.9513	−0.0602	0.1566
Dementia with Lewy bodies	PROT‐a‐2749 (SLC25A18)	5.1518	0.8209		
Dementia with Lewy bodies	PROT‐a‐300 (C1QBP)	13.1909	0.7801	0.0016	0.9588
Dementia with Lewy bodies	PROT‐a‐300 (C1QBP)	13.1936	0.8285		
Dementia with Lewy bodies	PROT‐a‐308 (C21orf33)	8.0360	0.7101	0.0268	0.5937
Dementia with Lewy bodies	PROT‐a‐308 (C21orf33)	8.3378	0.7582		

### 3.7 Reverse MR

**FIGURE 9:**
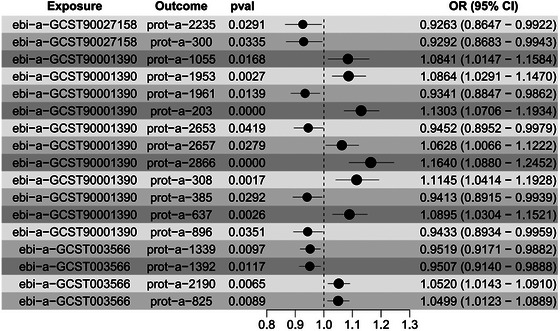
Reverse Mendelian randomization (MR) analysis results shown as the forest plot, showing significant associations between the mitochondrial protein levels and neurological diseases. OD, odds ratio.

Reverse MR analysis was conducted with neurodegenerative disease‐associated genes as exposure factors and mitochondrial proteins as outcome factors (Figure 9). The results revealed significant associations between the mitochondrial protein levels and AD ebi‐a‐GCST90027158, dementia ebi‐a‐GCST90001390, and multiple sclerosis ebi‐a‐GCST003566. Specifically, a bidirectional causal relationship was observed between AD and the PROT‐a‐308 (C21orf33) protein. Further research is required to determine whether third‐party regulations are involved.

## 4 Discussion

Neurodegenerative diseases include a group of disorders characterized by progressive dysfunction and neuronal loss leading to debilitating cognitive and motor impairments. Diseases such as AD, Parkinson's disease, ALS, and Huntington's disease pose significant challenges owing to their increasing prevalence and profound impact on the quality of life. Despite advancements in therapeutic approaches targeting various molecular pathways, clinical trials have commonly reported suboptimal outcomes in halting disease progression or improving cognitive decline. Treatments for neurodegenerative diseases such as AD and PD typically have limited effectiveness (Honig et al. 2018, Parkinson Study Group STEADY‐PD III Investigators 2020, Sperling et al. 2023). Mitochondrial dysfunction is a hallmark of many neurodegenerative diseases and affects cellular energy metabolism and oxidative stress pathways, which are crucial for neuronal survival. Therapeutic strategies aimed at enhancing mitochondrial biogenesis and functions are promising in preclinical models (Sorrentino et al. 2017, Zhao et al. 2011). As such, the role of the mitochondria in neurodegenerative diseases has garnered increasing attention.

In the present study, we analyzed the causal relationships between mitochondrial proteins and neurodegenerative diseases using a bidirectional MR approach. We confirmed, for the first time, that genetic mutations in mitochondrial proteins are causally linked to the onset of neurodegenerative diseases. These results indicate a significant association between mitochondrial protein mutations and multiple neurodegenerative diseases, highlighting the crucial role of mitochondrial proteins in their development. Some of our findings are consistent with those of previous studies. We found that mutations in FARS2 positively correlated with susceptibility to AD, which was supported by consistent validation across datasets. Chen et al. (2022) previously demonstrated that Fars2 defects affect neuronal development and enhance neuronal apoptosis by impairing mitochondrial function. Additionally, we observed that genetic mutations in LRPPRC and MUL1 increased susceptibility to PD, possibly via action on mitochondrial autophagy. Previous studies have shown that LRPPRC reduces BCL‐2 levels and releases more beclin 1, leading to the activation of autophagy through the beclin 1‐PI3KCIII complex (Zou et al. 2013). MUL1 is a multifunctional mitochondrial membrane protein that functions partly through its E3 ubiquitin ligase activity to ubiquitinate and degrade MFN2. Neurons lacking MUL1 function exhibit enhanced recruitment of fragmented mitochondria and increased mitochondrial autophagy under mild mitochondrial stress. Conversely, MUL1 overexpression inhibits PRKN translocation and mitochondrial autophagy (Puri et al. 2020). These hypotheses require further validation in PD‐related studies.

Genetic mutations in the same protein may play opposing roles in different diseases. For example, we found that mutations in C1QBP (a protein which regulates mitochondrial biogenesis and stress responses, ensuring the integrity and function of mitochondria under physiological and pathological conditions) increased the susceptibility to MS but conferred protection against ALS and LBD. This suggests the need for further exploration of the specific functional changes in proteins after mutations at different loci, as well as the detailed cellular biological functions of the same protein in different neurodegenerative diseases.

Furthermore, we conducted reverse MR analysis focusing on neurodegenerative disease‐related genes as exposures and mitochondrial proteins as outcomes. Our results revealed significant associations between mitochondrial protein levels and AD, Lewy body dementia, and multiple sclerosis. Interestingly, C21orf33 showed a bidirectional causal relationship with Lewy body dementia. This raises the intriguing possibility of third‐party mediation between C21orf33 and Lewy body dementia, which warrants further investigation.

In summary, our study represents the first large‐scale evaluation of the genetic associations between mitochondrial proteins and neurodegenerative diseases using bidirectional MR analysis and extensive GWAS data. By effectively controlling confounding factors and reverse causation bias, we were able to clarify the causal relationship between mitochondrial proteins and these diseases. Our findings advance the understanding of the mitochondrial roles in neurodegenerative diseases and underscore the potential of mitochondria‐targeted therapies for slowing disease progression and improving patient outcomes.

In conclusion, our study advances the understanding of the role of mitochondria in neurodegenerative diseases and highlights the potential of mitochondria‐targeted therapies to slow disease progression and enhance patient outcomes.

## 5 Conclusion

This study used bidirectional MR to explore the causal relationships between mitochondrial proteins and various neurodegenerative diseases. The results demonstrated significant correlations between genetic mutations in mitochondrial proteins and multiple neurodegenerative diseases, thus highlighting the critical role of mitochondrial proteins in the pathogenesis of these conditions. Additionally, reverse transcription studies have identified correlations between several neurodegenerative diseases and mitochondrial proteins. These findings provide new insights into the genetic relationships between mitochondrial proteins and neurodegenerative diseases and offer promising clues for the prevention and treatment of related disorders. However, further research is required to validate these results and to delve deeper into the underlying biological mechanisms.

## Author Contributions

**Fangyuan Wang**: software, data curation, formal analysis, writing–original draft, writing–review and editing, methodology, visualization. **Zhou Jing**: software, formal analysis, data curation, writing–original draft. **Qingyi Wang**: data curation. **Minghe Li**: data curation. **Bingqi Lu**: data curation. **Ao Huo**: data curation. **Chenglin Zhao**: data curation. **Huanyu Zhou**: data curation. **Wulong Liang**: supervision, writing–review and editing, resources. **Weihua Hu**: supervision, resources, writing–review and editing. **Xudong Fu**: supervision, funding acquisition, writing–review and editing, resources.

## Conflicts of Interest

The authors declare no conflicts of interest.

### Peer Review

The peer review history for this article is available at https://publons.com/publon/10.1002/brb3.70283.

## Supporting information



Table S1 Proteins related to mitochondria.
Table S2 Neurodegenerative disease‐related GWAS data.

## Data Availability

The data that support the findings of this study are available in the GWAS Catalog at https://www.ebi.ac.uk/gwas/, and IEU OpenGWAS at https://gwas.mrcieu.ac.uk/.
