# Supplementary material for: Bidirectional Mendelian Randomization Analysis of the Association Between Mitochondrial Proteins and Neurodegenerative Diseases
Source: Brain Behav. 2025 Jan 20;15(1):e70283. doi: 10.1002/brb3.70283 (PMC11744296; doi:10.1002/brb3.70283)
Supplement: Supplementary file 1 — Table S1 Proteins related to mitochondria. Table S2 Neurodegenerative disease‐related GWAS data. [file BRB3-15-e70283-s001.docx]

SUPPORTING INFORMATION

Table S1 Proteins related to mitochondria

| Trait | Genesymble | GWAS ID |
| --- | --- | --- |
| Phenylalanine–tRNA ligase, mitochondrial | FARS2 | PROT-a-1055(FARS2) |
| Glutaredoxin-2, mitochondrial | GLRX2 | PROT-a-1220(GLRX2) |
| GrpE protein homolog 1, mitochondrial | GRPEL1 | PROT-a-1281(GRPEL1) |
| Histidine triad nucleotide-binding protein 2, mitochondrial | HINT2 | PROT-a-1339(HINT2) |
| Hydroxymethylglutaryl-CoA synthase, mitochondrial | HMGCS2 | PROT-a-1356(HMGCS2) |
| 4-hydroxy-2-oxoglutarate aldolase, mitochondrial | HOGA1 | PROT-a-1368(HOGA1) |
| Serine protease HTRA2, mitochondrial | HTRA2 | PROT-a-1392(HTRA2) |
| Iron-sulfur cluster assembly enzyme ISCU, mitochondrial | ISCU | PROT-a-1572(ISCU) |
| Lon protease homolog, mitochondrial | LONP1 | PROT-a-1761(LONP1) |
| Leucine-rich PPR motif-containing protein, mitochondrial | LRPPRC | PROT-a-1783(LRPPRC) |
| Methylmalonyl-CoA epimerase, mitochondrial | MCEE | PROT-a-1864(MCEE) |
| Malonyl-CoA decarboxylase, mitochondrial | MLYCD | PROT-a-1907(MLYCD) |
| 39S ribosomal protein L14, mitochondrial | MRPL14 | PROT-a-1940(MRPL14) |
| 39S ribosomal protein L32, mitochondrial | MRPL32 | PROT-a-1941(MRPL32) |
| 39S ribosomal protein L33, mitochondrial | MRPL33 | PROT-a-1942(MRPL33) |
| 39S ribosomal protein L34, mitochondrial | MRPL34 | PROT-a-1943(MRPL34) |
| 39S ribosomal protein L52, mitochondrial | MRPL52 | PROT-a-1944(MRPL52) |
| Ribosome-recycling factor, mitochondrial | MRRF | PROT-a-1945(MRRF) |
| Mitochondrial peptide methionine sulfoxide reductase | MsrA | PROT-a-1953(MsrA) |
| Mitochondrial fission regulator 1 | MTF1 | PROT-a-1961(MTF1) |
| Poly(A) RNA polymerase, mitochondrial | MTPAP | PROT-a-1964(MTPAP) |
| Peptide chain release factor 1-like, mitochondrial | ICT1 | PROT-a-1965(ICT1) |
| Mitochondrial ubiquitin ligase activator of NFKB 1 | MUL1 | PROT-a-1969(MUL1) |
| Mitochondrial ubiquitin ligase activator of NFKB 1 | MUL1 | PROT-a-1970(MUL1) |
| N-acetylglutamate synthase, mitochondrial | NAGS | PROT-a-1997(NAGS) |
| NADH dehydrogenase [ubiquinone] 1 beta subcomplex subunit 11, mitochondrial | NDUFB11 | PROT-a-2022(NDUFB11) |
| NADH dehydrogenase [ubiquinone] 1 beta subcomplex subunit 8, mitochondrial | NDUFB8 | PROT-a-2024(NDUFB8) |
| NADH dehydrogenase [ubiquinone] iron-sulfur protein 4, mitochondrial | NDUFS4 | PROT-a-2025(NDUFS4) |
| NADH dehydrogenase [ubiquinone] flavoprotein 2, mitochondrial | NDUFV2 | PROT-a-2026(NDUFV2) |
| ATP synthase subunit beta, mitochondrial | ATP5B | PROT-a-203(ATP5B) |
| NFU1 iron-sulfur cluster scaffold homolog, mitochondrial | NFU1 | PROT-a-2041(NFU1) |
| Nucleoside diphosphate-linked moiety X motif 8, mitochondrial | NUDT8 | PROT-a-2128(NUDT8) |
| ADP-ribose pyrophosphatase, mitochondrial | NUDT9 | PROT-a-2129(NUDT9) |
| Pyruvate carboxylase, mitochondrial | PC | PROT-a-2190(PC) |
| [Pyruvate dehydrogenase (acetyl-transferring)] kinase isozyme 1, mitochondrial | PDK1 | PROT-a-2235(PDK1) |
| [Pyruvate dehydrogenase (acetyl-transferring)] kinase isozyme 2, mitochondrial | PDK2 | PROT-a-2236(PDK2) |
| tRNA pseudouridine synthase A, mitochondrial | TRUB1 | PROT-a-2454(TRUB1) |
| Oligoribonuclease, mitochondrial | REXO2 | PROT-a-2526(REXO2) |
| rRNA methyltransferase 3, mitochondrial | MRMT3 | PROT-a-2575(MRMT3) |
| Serine–tRNA ligase, mitochondrial | SARS2 | PROT-a-2627(SARS2) |
| Protein SCO1 homolog, mitochondrial | SCO1 | PROT-a-2653(SCO1) |
| Succinate dehydrogenase assembly factor 2, mitochondrial | SDHAF2 | PROT-a-2657(SDHAF2) |
| NAD-dependent protein deacylase sirtuin-5, mitochondrial | SIRT5 | PROT-a-2737(SIRT5) |
| Mitochondrial glutamate carrier 2 | SLC25A18 | PROT-a-2749(SLC25A18) |
| Mitochondrial sodium/hydrogen exchanger 9B2 | NHA2 | PROT-a-2764(NHA2) |
| Essential MCU regulator, mitochondrial | MICU1 | PROT-a-2776(MICU1) |
| Superoxide dismutase [Mn], mitochondrial | SOD2 | PROT-a-2799(SOD2) |
| Steroidogenic acute regulatory protein, mitochondrial | STAR | PROT-a-2866(STAR) |
| Complement component 1 Q subcomponent-binding protein, mitochondrial | C1QBP | PROT-a-300(C1QBP) |
| Transmembrane protein 70, mitochondrial | TMEM70 | PROT-a-3015(TMEM70) |
| ES1 protein homolog, mitochondrial | C21orf33 | PROT-a-308(C21orf33) |
| Carbonic anhydrase 5A, mitochondrial | CA5A | PROT-a-332(CA5A) |
| Coiled-coil domain-containing protein 90B, mitochondrial | CCDC90B | PROT-a-385(CCDC90B) |
| Coiled-coil-helix-coiled-coil-helix domain-containing protein 10, mitochondrial | CHCHD10 | PROT-a-534(CHCHD10) |
| Cytochrome c oxidase assembly factor 3 homolog, mitochondrial | COA3 | PROT-a-612(COA3) |
| Apoptosis-inducing factor 1, mitochondrial | AIFM1 | PROT-a-63(AIFM1) |
| Cytochrome c oxidase subunit 4 isoform 2, mitochondrial | COX4I2 | PROT-a-637(COX4I2) |
| Cytochrome c oxidase subunit 5B, mitochondrial | COX5B | PROT-a-638(COX5B) |
| Apoptosis-inducing factor 1, mitochondrial | AIFM1 | PROT-a-64(AIFM1) |
| Cytochrome c oxidase subunit 7A1, mitochondrial | COX7A1 | PROT-a-640(COX7A1) |
| Cytochrome c oxidase subunit 8A, mitochondrial | COX8A | PROT-a-641(COX8A) |
| Diablo homolog, mitochondrial | DIABLO | PROT-a-818(DIABLO) |
| Dihydrolipoyl dehydrogenase, mitochondrial | DLD | PROT-a-825(DLD) |
| Mitochondrial import inner membrane translocase subunit TIM14 | TIMM14 | PROT-a-847(TIMM14) |
| Calcium uptake protein 3, mitochondrial | MICU3 | PROT-a-896(MICU3) |
| Persulfide dioxygenase ETHE1, mitochondrial | ETHE1 | PROT-a-992(ETHE1) |

Table S2 Neurodegenerative disease-related GWAS data

| **GWAS ID** | **Year** | **Trait** | **Sample size** | **Number of SNPs** |
| --- | --- | --- | --- | --- |
| ebi-a-GCST90027158 | 2022 | Alzheimer disease | 487,511 | 20,921,626 |
| ebi-a-GCST002245 | 2013 | Alzheimer disease（test） | 55,134 | 7,022,150 |
| finn-b-G6_PARKINSON | 2021 | Parkinson’s disease | 218,792 | 16,380,466 |
| ebi-a-GCST90018894 | 2021 | Parkinson’s disease（test） | 480,018 | 24,194,622 |
| ebi-a-GCST90027163 | 2021 | Amyotrophic lateral sclerosis | 138,086 | 10,426,600 |
| ebi-a-GCST003566 | 2016 | Multiple sclerosis | 15,283 | 7,910,365 |
| ebi-a-GCST90001390 | 2021 | Dementia with Lewy bodies | 6,618 | 7,593,175 |
